# Early Blindness Limits the Head-Trunk Coordination Development for Horizontal Reorientation

**DOI:** 10.3389/fnhum.2021.699312

**Published:** 2021-07-16

**Authors:** Davide Esposito, Alice Bollini, Monica Gori

**Affiliations:** ^1^Unit for Visually Impaired People, Istituto Italiano di Tecnologia, Genoa, Italy; ^2^DIBRIS, Università di Genova, Genoa, Italy

**Keywords:** audiomotor integration, egocentric reference frame, head-trunk coordination, early blindness, virtual reality, subjective straight-ahead

## Abstract

During locomotion, goal-directed orientation movements in the horizontal plane require a high degree of head-trunk coordination. This coordination is acquired during childhood. Since early visual loss is linked to motor control deficits, we hypothesize that it may also affect the development of head-trunk coordination for horizontal rotations. However, no direct evidence exists about such a deficit. To assess this hypothesis, we tested early blind and sighted individuals on dynamic sound alignment through a head-pointing task with sounds delivered in acoustic virtual reality. Participants could perform the head-pointing with no constraints, or they were asked to immobilize their trunk voluntarily. Kinematics of head and trunk were assessed individually and with respect to each other, together with spatial task performance. Results indicated a head-trunk coordination deficit in the early blind group; yet, they could dampen their trunk movements so as not to let their coordination deficit affect spatial performance. This result highlights the role of vision in the development of head-trunk coordination for goal-directed horizontal rotations. It also calls for clarification on the impact of the blindness-related head-trunk coordination deficit on the performance of more complex tasks akin to daily life activities such as steering during locomotion or reaching to targets placed sideways.

## Introduction

How we move is closely linked to how we perceive. This connection is supported by several experimental pieces of evidence showing abnormal motor patterns in people with different types of sensory disabilities, like visual (Haibach et al., 2014), hearing (Vitkovic et al., 2016), and vestibular (Inoue et al., 2013) impairments. However, such evidence refers to passive postural balance and gross-motor abilities that involve the control of limbs. Much less is known about the influence of sensory impairments on the coordination of head and trunk goal-oriented movements. Yet, head-trunk coordination is of primary importance for accomplishing fundamental tasks, such as moving (re-orienting) to new target locations (Hollands et al., 2001). In fact, it has been shown that head-only rotations are used by the brain to prompt the body to steer toward the new head direction during locomotion (Patla et al., 1999). Interestingly, it is believed this motor pattern provides the brain with a stable spatial reference frame for body reorientation (Grasso et al., 1996).

Despite its importance, the coordination of head and trunk movements does not seem innate but is instead acquired through development. Assaiante et al. (2005) showed that children up to five-years-old move head and trunk together to some degree in postural, locomotion, and reaching tasks (Assaiante, 1998). In the same study, the researchers named this motor behavior *“en-bloc,”* in contrast to the adults’ “articulated” strategy introduced above, where head and trunk unbind and move independently. Moving head and trunk together as children do is supposed to simplify motor control by reducing the number of degrees of motion involved in a movement (Assaiante et al., 2005). Nevertheless, a significant amount of information for one’s horizontal orientation estimation is provided by proprioceptive inputs from the neck (Warren, 1998; Pettorossi and Schieppati, 2014); so, turning head and trunk together instead of coordinating the two body parts would impoverish that information source. If the available information for horizontal orientation estimation is poorer, one can hypothesize that the estimation quality will be poorer too.

Given that sensory disabilities affect several aspects of motor development, and given the developmental nature of head-trunk coordination, sensory impairments may affect head-trunk coordination as well. For visual loss, this is certainly the case. As infants, totally blind individuals show a clear delay in head control development (Prechtl et al., 2001). As adults, blind people lock head and trunk movements in postural balance (Easton et al., 1998; Schmid et al., 2007; Alotaibi et al., 2016). Such results recall the abovementioned “en-bloc” strategy, typical of sighted children whose head-trunk coordination is incomplete. Sighted children use this strategy in both postural balance and horizontal orienting movements (Assaiante et al., 2005); therefore, such coupling may hold in early blind adults too. If such a strategy were used by the adult blind population for horizontal reorientation, it should reduce proprioceptive inputs from the neck. One may therefore expect blind people to perform poorly in tasks that require horizontal head turns, such as horizontal sound localization by head pointing. Instead, scientific evidence shows that they perform as well as, or even better than, sighted individuals in this task (Lessard et al., 1998; Röder et al., 1999; Collignon et al., 2009; Lewald, 2013).

The apparent contradiction between spatial and kinematic performance among blind people may be explained in two ways. On the one hand, visual loss does not cause head-trunk coordination issues for goal-directed horizontal rotation. On the other hand, head-trunk coordination may be impaired in blindness, but simple head-pointing tasks with none or passive-only constraints on trunk movements may not challenge head-trunk coordination enough to affect spatial performance. However, to the best of our knowledge, no prior study has directly tested blind people’s head-trunk coordination in goal-directed horizontal rotations, nor their horizontal audio-spatial performance while their head-trunk coordination is challenged. Given that early blind adults show child-like head-trunk coordination for postural tasks (Easton et al., 1998; Schmid et al., 2007; Alotaibi et al., 2016), and given that in children the head-trunk coordination strategy is similar in postural tasks and in tasks requiring goal-directed horizontal rotations (Assaiante et al., 2005), we hypothesize the early blind adults would also show child-like head-trunk coordination in a goal-directed horizontal rotation task.

We developed a dynamic sound alignment task on an acoustic virtual reality (AVR) platform made expressly to test this hypothesis. AVR environments are handy tools for defining complex tasks involving auditory localization in the horizontal plane because they give results similar to those with real speakers (Wenzel et al., 1993). Furthermore, they provide portable setups that guarantee more control over the sound position relative to the ears and inherently provide kinematic data about the tracked body parts. In order to test our hypothesis, AVR allowed us to define an experimental task based on sound localization in the horizontal plane via dynamic head-pointing, with or without acoustic feedback for trunk movements. With their kinematic profiles, we could directly evaluate participants’ head-trunk coordination. At the same time, with the sound localization task we could check the extent to which different degrees of head-trunk coordination relate to audio-spatial performance. In order to challenge participants’ head-trunk coordination, we set a head-trunk coordination constraint to demand voluntary trunk immobilization by means of acoustic feedback for trunk movements and explicit instructions. This condition was paired with another, where the head-trunk coordination was spontaneous, without feedback. Doing so, we could expose behavioral differences in horizontal sound alignment by head-pointing with and without demanding head-trunk coordination.

We tested typical sighted and early blind participants on our AVR platform. Following our hypothesis we predicted that if early visual deprivation affected the head-trunk coordination for horizontal rotations, early blind people would differ from sighted controls when demanded to coordinate head and trunk in kinematic behavior and, if the impairment is large enough, also in spatial performance.

## Methods and Analysis

### Participants

In total, 21 individuals, 10 congenital blind (3 males, 7 females, age = 33.2 ± 3.19 years old, the clinical details of the participants’ pathologies are reported in Table 1) and 11 sighted individuals (6 males, 5 females, age = 31.27 ± 3.92 years old) were involved in the study. All of them were enrolled by local contacts in Genoa. Informed consent was obtained from all participants. The study followed the Helsinki Declaration’s tenets and was approved by the ethics committee of the local health service (Comitato Etico, ASL 3, Genova).

**TABLE 1:** Clinical details of blind participants.

	**Gender**	**Age**	**Pathology**	**Blindness onset**	**Residual vision**
P1	F	32	Retinopathy	Before birth	No vision
P2	F	20	Retinopathy	Before birth	Lights and shadows
P3	F	29	Retinopathy	Before birth	No vision
P4	M	27	Leber’s amaurosi	Since birth	No vision
P5	F	26	Glaucoma and retinal detachment	Before birth	No vision
P6	M	46	Leber’s disease	Before birth	No vision
P7	M	52	Unknown	Before birth	Lights and shadows
P8	F	30	Retinitis pigmentosa	Since birth	Lights and shadows
P9	F	28	Microphtalmia	Since birth	No vision
P10	F	42	Retinopathy	Since birth	No vision

### Physical Experimental Setting

**FIGURE 1:**
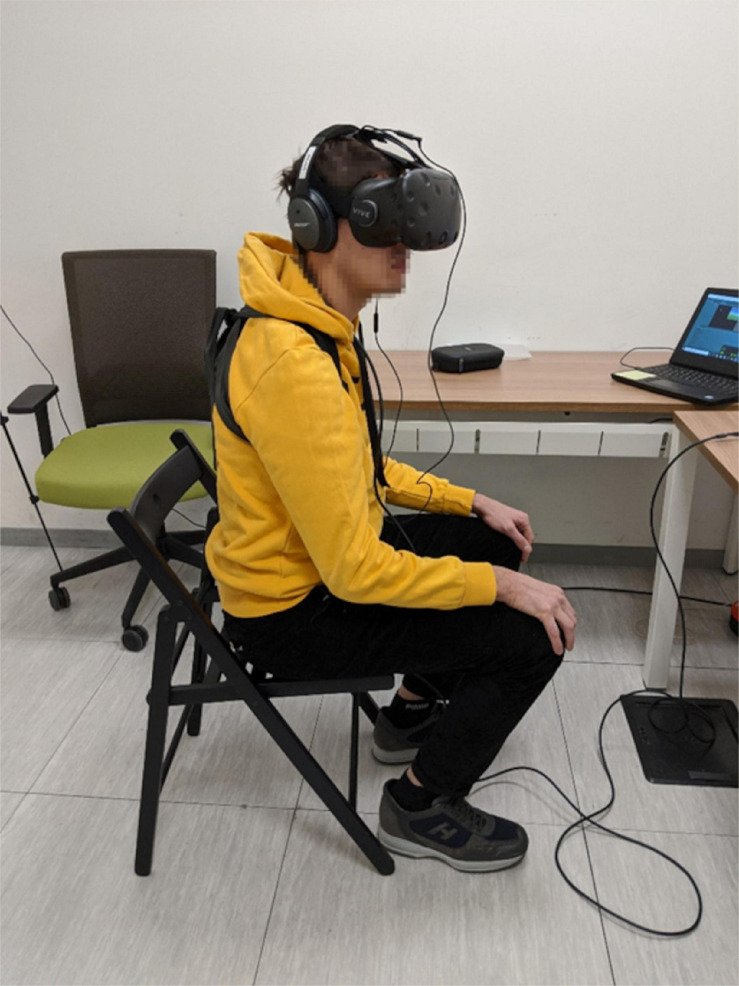
Hardware and room setting during an experimental session. The participant wears the VR headset, the headphones and a custom-made harness to keep the smartphone on his/her back. During each trial, the participant sits and his/her back is free to rotate.

The AVR environment created for this experiment was developed with the game engine Unity 3D. The spatial blending of sounds was made using the *resonance* package (Google, 2018). The sound was delivered via commercially available BOSE^®^ over-ear headphones. For the purpose of the kinematic assessment, participants’ head and trunk movements were tracked. The participants’ head rotations were tracked by the head-mounted display (HMD) itself, at a sampling rate of 90 Hz, which is the frequency of Unity’s main loop. The trunk rotations were tracked by an LG^®^ google nexus 4 smartphone used as a wireless inertial measurement unit thanks to the app HyperIMU (Ianovir, 2019), with a nominal sampling rate of 100 Hz. Both sensors have a resolution of 0.1°. The incoming samples were asynchronously collected via an User Datagram Protocol (UDP) socket, stacked and averaged at the frequency of 90 Hz. The smartphone was fixed to participants’ backs using a custom-made harness. In the context of AVR, the HMD screens were blank, so the virtual reality headset (HTC^®^ VIVE) was uniquely used to track the participants’ head movements. During the experiment, participants were seated and their trunk was free to rotate (Figure 1). Unity’s refresh rate of the physics engine was kept at the standard value of 30 Hz to maintain a good tradeoff between performance and computational cost. The HTC Vive headset was chosen because its tracking system is reliable, accurate, validated for scientific research (Le Chénéchal and Chatel-Goldman, 2018; Niehorster et al., 2017; Verdelet et al., 2019) and comes with default Unity 3D integration. The smartphone IMU was preferred over HTC Vive trackers to reduce the risk of tracking loss for whatever reason, which is a known performance issue for the Vive system (Niehorster et al., 2017; Verdelet et al., 2019). IMUs, instead, are subject to drift. Recalibration was performed before starting a new condition and after approximately 10 trials to compensate for the drift effect.

### Virtual Experimental Setting

**FIGURE 2:**
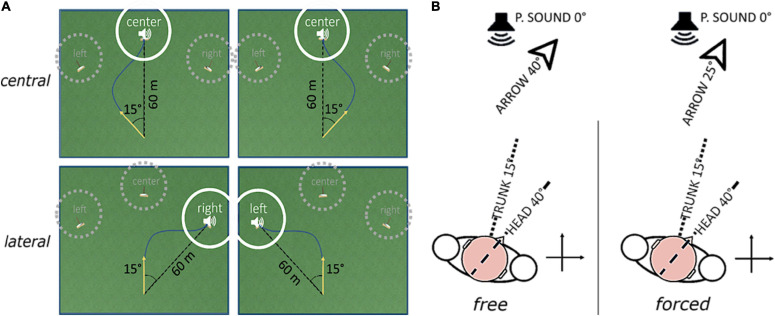
Schematic description of how the experimental factors “direction” **(A)** and “coordination” **(B)** define the game condition. Panel **(A)** shows the virtual playfield and two examples of possible arrow trajectories (dark blue line) for each “direction” level. The yellow arrow highlights the starting arrow direction. Targets in dotted gray circles are inactive during the trial; the only active target is the one in full white circle. Examples on the same column show conditions where the relative position of arrow and target is the same, but the absolute target position (i.e., the “direction” level) is different. Panel **(B)** shows the relationship between body positions, arrow direction and perceived source position according to each “coordination” level. Head yaw, trunk yaw, and arrow direction are highlighted by, respectively, a dotted line and a dashed line. P.SOUND means perceived sound position. With forced coordination, immobilization of the trunk is demanded, and the arrow direction is given by the difference between head and trunk rotations.

The AVR platform we developed defines four goal-directed steering tasks in a first-person perspective, described as archery-like games, based on the same virtual environment. The virtual environment’s absolute reference frame is aligned to the participant’s seat via the standard calibration phase for the HTC VIVE. The unit of measure of length is the unity unit (uu). Objects’ sizes and distances have been designed to match the uu with the meter; therefore, the meter will be used to describe the spatial parameters. The camera view is 1.7 m above the ground and its position in the virtual environment mimics the arrow position. The arrow can be in two states only: loaded and shot. In the loaded state, the arrow appears at the origin of the virtual environment’s absolute reference frame; it does not move, but it can rotate around the vertical axis. The transition from loaded to shot state is automatic. It happens when the arrow orientation lies inside a trigger window for a time span randomly chosen from 1 to 3 s. The arrow, once shot, advances at a fixed velocity of 10 $\frac{m}{s}$ in the horizontal plane. The target is a source of intermittent pink noise with 5 Hz duty cycle; its spatial attenuation follows an isotropic logarithmic function. It is 60 m distant from the starting point and can appear at three absolute angles: −15°, 0°, +15°. Regardless of the task, trigger window, and target centers are always shifted ± 15° apart from each other. Figure 2A displays the virtual environment, together with the set of possible trigger window-target geometrical configurations.

### Tasks Description

The four above-mentioned archery-like, first-person perspective, goal-directed steering tasks are actually four conditions of one base task, derived by four different parameterizations. The base task consists of leading the arrow, whose initial trajectory is by-design 15° apart from the target, toward the target itself. The arrow flight direction depends on a combination of head and trunk rotations around the vertical axis, and the participants’ goal is to hit the target center.

The conditions are defined according to two factors: “direction” and “coordination” (Figure 2).

The factor “coordination” rules the combination of head and trunk movements leading the arrow direction in the virtual space. Its two levels are *free* and *forced.* In the *free* level, the experiment baseline, the arrow rotation mimics the head yaw uniquely and participants are told by the experimenter to move freely, only caring about hitting the target. In the *forced* condition, the test, the trunk is used as virtual environment’s reference frame. In this way, the arrow rotation mimics the difference between head and trunk yaws, and the relative target-to-trunk rotation is kept constant throughout the trial. This condition was designed to discourage trunk rotations implicitly. Moreover, participants were explicitly asked by the experimenter to voluntarily immobilize their trunk as much as they can. The comparison between these two levels, one without coordination demand and the other with active coordination demand, is the method used to assess the relationship between early visual impairment and head-trunk coordination.

The factor “direction” sets the relative initial position of the target concerning the arrow. Its two levels are *central* and *lateral*. In the *central* level, the target is aligned with the participant’s straight-ahead and the starting position of the arrow is 15° to the side, randomly alternated between rightward and leftward. In the *lateral* level, the target is positioned at 15° from the participant’s straight-ahead, randomly alternated between rightward and leftward, and the starting position of the arrow is 0° (the participant starts with head and trunk aligned to the straight-ahead direction). The distinction between *central* and *lateral* targets was implemented because previous research on auditory localization in the horizontal plane by head-pointing showed better spatial performance in frontal than in eccentric stimuli (Makous and Middlebrooks, 1990; Lewald et al., 2000; Occhigrossi et al., 2021). By doing so, we aimed at evaluating possible differences in motor behavior that could explain those findings.

Each trial is made of two steps: positioning and execution. In the first step, positioning, the arrow is yet to be shot and the participant places the arrow in the trigger as mentioned above the window. Acoustic feedback is provided to help participants find the required starting orientation. This is an intermittent pure tone whose pitch is tuned by the angular distance between arrow and trigger window; the shorter the distance, the higher the pitch. The arrow shooting is announced by the interruption of the intermittent pure tone feedback and the reproduction of an arrow-shot-like sound. Data collected in this first step are not in the interest of this study. The second step, execution, is where the arrow moves and the task is accomplished. The trial end is notified by a prerecorded soundclip of an arrow hitting a wall if the target is hit, or the sound of a windblow if the target is missed.

### Experimental Procedure

At the participants’ arrival, they were given the following instructions: “Imagine you are on an arrow. Once shot, it will fly at a constant speed and you will control its direction only by moving a part of your body as I will tell you. Your goal is to drive the arrow toward the sound you will hear, which will correspond to the target. There are three possible target positions.” Then, they were blindfolded and introduced to the virtual platform by tactile exploration of a scaled plastic model of the environment; the experimenter made them track with a finger four plausible arrow paths, two for each “direction” condition (e.g., blue curved lines in Figure 2A). To let the participants familiarize themselves with the platform, a very short training session was performed, made of no more than eight trials in the baseline condition, *free* coordination. To make sure participants could exploit the audio feedback, the experimenter guided the participants’ head movements by hand during the first three/four familiarization trials. In the remaining trials, participants tried to drive the arrow on their own. For the training, only one “direction” level was randomly chosen, counterbalanced among participants.

Each experiment consisted of four runs, in randomized order and counterbalanced among participants. Each run was made of twenty trials. Before proceeding with a run, the corresponding requirements were explained to the participant by the experimenter. Breaks were allowed at any time according to participants’ needs. The whole experiment lasted approximately 30 min with no breaks.

### Data Analysis

Two behavioral aspects were evaluated in this experiment: task-related performance and motor behavior. The task-related performance was evaluated by means of accuracy and precision in hitting the target center. The arrow hit-point distance from target center (i.e., final error) was attributed a sign according to a target-based coordinate system. Specifically, given that the line joining target and absolute coordinates’ origin splits the virtual environment in two hemispaces, if the arrow end-point lied in the same hemispace as its initial trajectory, it would be positive. Otherwise, it would be negative. Then, data distributions from each condition were tested for normality using the Lilliefors test (Lilliefors, 1967). For both the distributions’ high non-normality rate and the small groups’ sample sizes, we decided to use non-parametric statistics. Consequently, the accuracy was computed as the median of the by-trial final error, and the precision as the inter-quartile range (IQR) of the by-trial final error.

The motor behavior evaluation was based on the analysis of head and trunk yaws (deg) collected during trials’ execution step. Raw signals were acquired at Unity’s main loop refresh rate, which is 90 Hz only approximately. To compensate for sampling jitter and missing data points, the signals were resampled at 90 Hz, then smoothed using an 18-samples moving average window. Further analyses were performed on yaw jerks (variation of angular acceleration, deg⋅s^–3^) in each execution step. Two measures were used to evaluate the motor behavior: root mean square (RMS) of the trunk yaw jerk signal, in brief trunk RMS, used to quantify how much it was moved; and the amplitude of cross-correlation peak between head and trunk yaw jerks, in brief cross-peak, used to quantify the similarity between head and trunk movements. Again, the median was used to aggregate by-trial measures.

Since data points in kinematic signals were not independent and identically distributed, head and trunk yaw jerks were prewhitened before computing cross-correlation (Dean and Dunsmuir, 2016). The Supplementary Material contains a full methodology description.

Accuracy, precision, RMS and cross-peak were analyzed using a three-ways 2 × 2 × 2 repeated-measures ANOVA on aligned rank transformed data, and ART ANOVA (Wobbrock et al., 2011) with “group” as a between-subjects factor (blind and sighted), and “direction” (*central* and *lateral*) and “coordination” (*free* and *forced*) as within-subjects factors. *Post-hoc* comparisons were performed via Wilcoxon test for within-group comparisons and Mann-Whitney test for between-group comparisons. In case the ART ANOVA returned significant interaction effects, *post-hoc* comparisons were performed between the interaction levels with one main level in common, and *P* values were Bonferroni corrected. Standardized effect sizes were computed along with the unstandardized tests. Partial eta squared is provided as standardized effect size for the ART ANOVA. Rank biserial correlation and its confidence interval are provided as standardized effect size for non-parametric *post-hoc* tests.

Kinematic data resampling, smoothing and differentiation, prewhitening, estimation of cross-peaks and RMS were made with the software MATLAB r2020a. ART ANOVA and *post-hoc* analyses were made with the software R. ART ANOVA were made with the package ARTool (Wobbrock et al., 2011). The final dataset can be found in the Zenodo repository at the link http://doi.org/10.5281/zenodo.4707477. Raw or intermediate datasets generated during the current study and code used for the analysis are available from the corresponding author on reasonable request.

## Results

**FIGURE 3:**
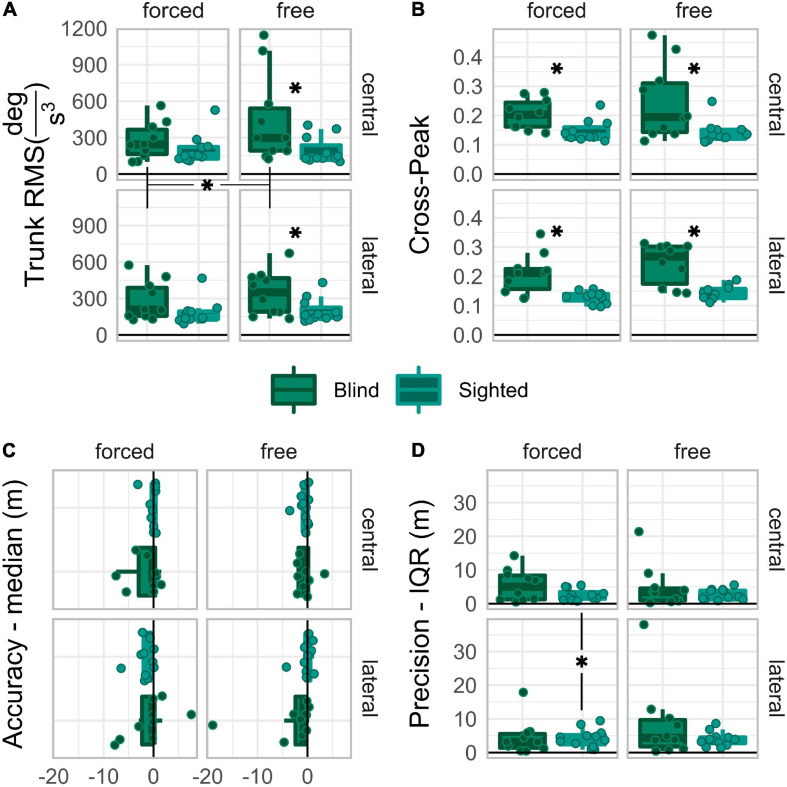
Boxplots of trunk RMS **(A)**, cross-peak **(B)**, Accuracy **(C)**, and Precision **(D)**, reported for each group and driving condition. Asterisks expose the *post-hoc* comparisons significances. Those between graphs’ labels expose the within-group main effects. Those in the space between subgraphs expose the 2-way within-group interaction effects. Those between boxplots in the same grid expose significant differences between groups.

Data collected with *forced* coordination were compared to those collected with *free* coordination to evaluate the ability of early blind individuals and sighted controls to localize dynamic sounds by head-pointing while self-immobilizing their trunk on both kinematics and spatial performance standpoints. The distinction between *central* and *lateral* direction levels was maintained in the analysis to assess behavioral differences between frontal and eccentric targets, as previously found for auditory localization by head-pointing (Lewald et al., 2000; Occhigrossi et al., 2021). Results are reviewed separately for each measure. Hypotheses for each measure are expressed in the corresponding subsection. All the results of the ANOVA tests are reported in Table 2. Data boxplots are reported in Figure 3.

**TABLE 2:** Results of the ANOVA tests for each of the four computed measures.

	**TRUNK JERK RMS**			**XCORR PEAKS**			**ACCURACY**			**PRECISION**		
**Effect**	**F(1,19)**	**Pr(> F)**	**p.eta^2^**	**F(1,19)**	**Pr(> F)**	**p.eta^2^**	**F(1,20)**	**Pr(> F)**	**p.eta^2^**	**F(1,20)**	**Pr(> F)**	**p.eta^2^**
group	18.40	0.000***	0.49	5.23	0.034*	0.22	0.39	0.539	0.02	0.19	0.665	0.01
direction	0.05	0.826	0.00	1.08	0.312	0.05	3.28	0.085	0.14	1.08	0.312	0.05
coordination	6.02	0.024*	0.24	12.02	0.003**	0.39	0.90	0.355	0.04	0.02	0.881	0.00
group:direction	0.32	0.578	0.02	1.71	0.206	0.08	0.41	0.527	0.02	0.00	0.980	0.00
group:coordination	3.59	0.074	0.16	9.04	0.007**	0.32	1.64	0.214	0.08	6.87	0.016*	0.26
direction:coordination	0.97	0.337	0.05	0.24	0.626	0.01	4.81	0.040*	0.19	0.46	0.506	0.02
group:direction:coordination	0.05	0.823	0.00	0.99	0.332	0.05	6.86	0.016*	0.26	9.54	0.006**	0.32

Significances are represented as following: *p ≤ 0.05, **p ≤ 0.01, ***p ≤ 0.001.

### Kinematics

Kinematic behavior was assessed in terms of trunk jerk RMS and cross-peak. The trunk jerk RMS is a measure of the amount of movement. A larger RMS means participants moved the trunk more. The cross-peak is a measure of similarity between the head and trunk angular jerk. A larger peak means more similar head and trunk movements.

#### Trunk Jerk RMS

If people tried to immobilize their trunk, their trunk RMS would be larger in *free* than in *forced* conditions; however, if early blind people struggled at it, their trunk RMS in *forced* conditions would be larger than that of sighted participants. If having targets straight-ahead facilitated trunk immobilization, the trunk RMS would be larger in the *lateral* than in the *central* direction. The ART ANOVA test reached significance for group and control main effects, *F_*group*_(1,19)* = 5.23, *p* = 0.034, *p.eta^2^_*group*_* = 0.22, *F_*control*_(1,19)* = 12.02, *p* = 0.003, *p.eta^2^_*control*_* = 0.39, *F_*direction*_(1,19)* = 1.08, *p* = 0.312, *p.eta^2^_*direction*_* = 0.05. Significance of the interaction effects was reached for “group:coordination,” *F(1,19)* = 9.04, *p* = 0.007, *p.eta^2^* = 0.32.

*Post-hoc* comparisons on the “group:coordination” interaction levels were performed under the alternative hypothesis that the trunk jerk RMS is larger in blind than in sighted participants, with *free* rather than with *forced* coordination. Figure 3C shows that the trunk jerk RMS was significantly larger in blind than in sighted participants only when the control condition was *free*, *U_*blind–sighted| free*_* = 88, *p* = 0.02, *r*_*rb*_ = 0.60, 95%CI [0.18,0.93], *U*_*blind–sighted| forced*_ = 75, *p* = 0.173, *r*_*rb*_ = 0.36, 95%CI [−0.15,0.84]. The RMS was significantly larger for the *free* than for the *forced* coordination level only in the blind group, *W*_*free–forced| blind*_ = 54, *p* = 0.004, *r*_*rb*_ = 0.96, 95%CI [0.64,1], while the sighted group did not show any significant control-related change, *W*_*free–forced| sighted*_ = 45, *p* = 0.32, *r_*rb*_* = 0.36, 95%CI [−0.24,1].

#### Cross-Peak

If early blind people could not immobilize their trunks, their cross-peaks would be higher than sighted participants, at least in the *forced* coordination level where sighted people are supposed to immobilize trunk movement. If having targets straight-ahead facilitated trunk immobilization, the cross-peaks would be larger in the *lateral* than in the *central* direction. The ART ANOVA test reached significance for group and control main effects only, *F_*group*_(1,19)* = 18.4, *p* < 0.001, *p.eta^2^_*group*_* = 0.49, *F_*control*_(1,19)* = 6.02, *p* = 0.024, *p.eta^2^_*control*_* = 0.24, *F_*direction*_(1,19)* = 0.05, *p* = 0.826, *p.eta^2^_*direction*_* = 0.00. No significance was reached in any interaction effect.

Even though no interaction effect reached significance, the “group:coordination” interaction approached significance and had a relatively large effect size, *F(1,19)* = 3.59, *p* = 0.074, *p.eta^2^* = 0.16. Therefore, *post-hoc* comparisons were performed on the levels of the “group:coordination” interaction, under the alternative hypothesis, that the cross-correlation peaks are larger in blind than in sighted individuals, and for *free* than for *forced* levels. Figure 3D shows that the crosscorrelation peaks were significantly larger in blind than in sighted participants regardless of control, *U_*blind–sighted| free*_* = 98, *p* = 0.002, *r_*rb*_* = 0.78, 95%CI [0.40,1], *U_*blind–sighted| forced*_* = 96, *p* = 0.003, *r_*rb*_* = 0.75, 95%CI [0.36,0.96], and that no significant difference held between *free* and *forced* in any group, *W_*free–forced| blind*_* = 45, *p* = 0.084, *r_*rb*_* = 0.64, 95%CI [−0.09,1] *W_*free–forced| sighted*_* = 43, *p* = 0.413, *r_*rb*_* = 0.30, 95%CI [−0.33,0.94].

To summarize, sighted people had small trunk RMS, unaffected by the coordination factor. Contrarily, blind people had significantly larger trunk jerk RMS with *free* than with *forced* coordination; early blind people had significantly larger trunk jerk RMS than sighted people only with *free* coordination. Moreover, early blind people had higher cross-correlation peaks than sighted individuals in every condition.

### Performance

The performance was assessed in terms of accuracy and precision. Accuracy was evaluated as the median of the by-trial final error. A negative value means a bias toward the hemifield of the initial direction. A positive value means bias away from the initial hemifield. Better accuracy means a value closer to zero. Precision was evaluated as IQR of the by-trial final error. Smaller IQR means better precision.

#### Accuracy

If the head-trunk coordination demand impaired participants’ accuracy in steering toward the target, they would be less accurate with *forced* than with *free* coordination. If having targets straight-ahead facilitated localization, accuracy would have more negative values in *lateral* than in *central* direction. The ART ANOVA test reached significance for none of the main effects, *F_*group*_(1,20)* = 0.19, *p* = 0.665, *p.eta^2^_*group*_* = 0.01, *F_*direction*_(1,20)* = 1.08, *p* = 0.312, *p.eta^2^_*direction*_* = 0.05, *F_*control*_(1,20)* = 0.02, *p* = 0.881, *p.eta^2^_*control*_* = 0.00. Significance of the interaction effects was reached for “group:coordination,” *F(1,20)* = 6.87, *p* = 0.016, *p.eta^2^* = 0.26, and “group:direction:coordination,” *F(1,20)* = 9.54, *p* = 0.006, *p.eta^2^* = 0.32.

*Post-hoc* comparisons on “group:direction:coordination” interaction levels were performed under the alternative hypothesis that accuracy is different between early blind and sighted, between *free* and *forced* levels, and more negative in *lateral* than in *central*. Figure 3A shows that none of the comparisons reached significance; however, the *central* vs. *lateral* comparison with *forced* coordination approached significance very closely in sighted, *W_*lateral–central| sighted:*__*forced*_* = 12, *p* = 0.051, *r_*rb*_* = −0.69, 95%CI [−1, −0.15], but not in blind individuals, *W_*lateral–central| blind:*__*forced*_* = 23, *p* = 1.000, *r_*rb*_* = −0.16, 95%CI [−0.85,0.6].

#### Precision

If the head-trunk coordination demand impaired participants’ precision in steering toward the target, they would be more precise with *free* than with *forced* coordination. If having targets straight-ahead facilitated localization, precision would be better in *central* than in *lateral* direction. The ART ANOVA test reached significance for none of the main effects, *F_*group*_(1,20)* = 0.39, *p* = 0.539, *p.eta^2^_*group*_* = 0.02, *F_*direction*_(1,20)* = 3.28, *p* = 0.085, *p.eta^2^_*direction*_* = 0.14, *F_*control*_(1,20)* = 0.9, *p* = 0.355, *p.eta^2^_*control*_* = 0.04. Significance was reached for the interaction effects “group:coordination,” *F(1,20)* = 1.64, *p* = 0.214, *p.eta^2^* = 0.08, and “group:direction:coordination,” *F(1,20)* = 6.86, *p* = 0.016 *p.eta^2^* = 0.26.

*Post-hoc* comparisons on the “group:direction:coordination” interaction levels were performed under the alternative hypothesis that precision is better in sighted than blind, in *central* than *lateral* direction, and with *free* than *forced* coordination. Figure 3B shows that sighted, not blind, were significantly more precise in the *central* than in the *lateral* direction when the control was *forced*, *W_*lateral–central| sighted:*__*forced*_* = 67, *p* = 0.040, *r_*rb*_* = 0.72, 95%CI [0.26,1], *W_*lateral–central| blind:*__*forced*_* = 22, *p* = 1.000, *r_*rb*_* = −0.20, 95%CI [−0.89,0.49].

To summarize, we could not find group-wise differences related to the head-trunk coordination demand. Moreover, only sighted people had better spatial performance when the target was straight-ahead than to the side, but only when trunk immobilization was demanded.

## Discussion

The goal of this study was to test the hypothesis that early visual loss impairs head-trunk coordination development for orienting movements in the horizontal plane. The investigation was performed by testing a group of early blind people on a head-pointing task with dynamic auditory stimuli delivered in AVR. In some trial blocks, a head-trunk coordination constraint was set implicitly and explicitly, inducing participants to immobilize their trunks. Kinematic behavior and spatial performance were assessed within-group by comparing trial blocks with and without coordination demand and between-group by comparing early blind participants with sighted blindfolded controls. The assessed aspects of kinematic behavior were similarity between head and trunk movements via cross-peak (head-trunk jerk cross-correlation peak amplitude) and trunk movement via trunk jerk RMS. The spatial performance was assessed by accuracy and precision: median and interquartile range of the distribution of the trial-by-trial final distances from the target center (Figure 3).

Two possible scenarios may have emerged. If early blindness did not impair head-trunk coordination for goal-directed horizontal rotation, no difference would have emerged in kinematic behavior or spatial performance. Alternatively, if early blindness impaired head-trunk coordination, early blind people would differ from sighted controls when demanded to coordinate head and trunk; this would definitely occur in their kinematic behavior and, if the impairment is large enough, also in spatial performance. Our results confirmed the hypothesis that early visual deprivation hampers the development of head-trunk coordination for orienting movements in the horizontal plane. Indeed, the early blind group showed an overall larger cross-peak than the blindfolded sighted group (Figure 3B). Moreover, when the trunk was free to move (*free* coordination), early blind people had larger trunk jerk RMS than sighted blindfolded in the same condition, and larger trunk jerk RMS when voluntary trunk immobilization was demanded (*forced* coordination) (Figure 3A). Altogether, the results concerning kinematic behavior describe the following scenario: early blind people, when free to rotate around the vertical axis, did so with head and trunk together; furthermore, when asked to immobilize their trunk, they did it by damping their trunk rotations (doing rotations with small amplitude) instead of avoiding them. Although the motor behavior exposed by the early blind group, hereafter named “damping” behavior, exposes some degree of head-trunk coordination, their head and trunk movements were more similar than those of sighted participants. The early blind group’s motor behavior recalled the “*en-bloc*” motor coordination strategy, typical of 3 to 8 year-old sighted children with incomplete head-trunk coordination development (Assaiante et al., 2005).

Several pieces of evidence, mainly from postural balance tasks (Easton et al., 1998; Schmid et al., 2007; Alotaibi et al., 2016), have suggested the existence of a link between early visual loss and head-trunk coordination deficit. Yet, to the best of our knowledge, this is the first study that directly identifies the connection for goal-directed orienting movements in the horizontal plane. The information obtained here is limited to acknowledging the existence of such a deficit; conclusions cannot be drawn about its etiology. Vision may be necessary for the development of head-trunk coordination, or it could act as the catalyst of a process driven, instead, by motor experience (Lopes et al., 2011). Indeed, visual disabilities are very often a great barrier for people to move freely (Marron and Bailey, 1982; Guralnik et al., 1994; West et al., 2002), and consequently limit their chances to explore and learn new motor commands, even the coordination of head and trunk.

One may expect a deficit in head-trunk coordination to have some consequences on spatial performance, at least when tasks require good motor control skills, i.e., the trial blocks with *forced* coordination. Our behavioral measures failed to identify differences between early blind and sighted groups (Figures 3C,D). This failure probably arises from the method’s inadequacy in causing the deficit to affect spatial performance: the implicit coordination demand was designed to affect performance if people could not immobilize their trunk. Early blind participants, instead, using “damping” behavior, managed to do so well enough that their performance was not affected. The “damping” behavior identified in this paper satisfactorily explains why early blind people perform as good as, or even better than sighted individuals, in horizontal sound localizations by head pointing (Lessard et al., 1998; Röder et al., 1999; Collignon et al., 2009; Lewald, 2013). Since generic head-pointing tasks with no or passive-only coordination constraints challenge head-trunk coordination less than our task, early blind people’s “damping” behavior compensates their head-trunk coordination deficit well enough not to let it affect head-pointing performance. It remains unclear whether the coordination deficit identified here can still cause performance drops in the unstructured setups found in everyday life, where motor and coordination demands are more complex and varied.

The comparison between trial blocks with targets in front of participants (*central*) versus those with targets to the side (*lateral*) provides another point of discussion in light of the literature on egocentric auditory localization. Past studies on the typical population have highlighted, using different methods, some kind of spatial performance drop when targets were placed at eccentric positions concerning the participants’ straight-ahead and when the head was turned (Makous and Middlebrooks, 1990; Lewald et al., 2000; Occhigrossi et al., 2021). Most of these studies identified a stimulus eccentricity underestimation bias, that is, accuracy loss. It was also shown that early blind people did not exhibit the underestimation bias and, therefore, obtained more accurate results than sighted people (Lessard et al., 1998; Zwiers et al., 2001; Lewald, 2013). Our results contribute to such body of evidence by identifying a precision, not accuracy, drop in sighted participants, only with *forced* coordination. At first glance, our results may appear in contrast with the previously identified accuracy drop in head-pointing localization. They complement the previous findings by showing what happens in a less structured context. The bias identified in head-pointing tasks was shown to be a function of head-on-trunk eccentricity (Makous and Middlebrooks, 1990; Lewald et al., 2000; Occhigrossi et al., 2021). Instead, in our task, the relative position between participant’s seat and sound source is continuously updated (participants “sit” on the virtual arrow, which advances in the virtual space at fixed speed). By doing so, each trial ends with a different head eccentricity. In our case, it is likely that the aggregation of trials that contain different head eccentricities, hence different biases, resulted in more dispersed samples. In support of this view is the fact that the pattern of our results on precision strictly matches the previous literature for accuracy when the coordination is *forced*, that is when the trunk is voluntarily immobilized and head-on-trunk rotations are maximized.

To conclude, early visual deprivation affects the full development of head-trunk coordination for orienting movements in the horizontal plane, yet the degree of control over the trunk obtained without early visual experience is enough to dampen unwanted trunk rotations. This “damping” strategy lets early blind people perform head-pointing tasks unaffected, even when sounds are not static and coordination constraints are demanded. The etiology of this deficit remains unclear and will be the object of further investigation, as well as the impact of the coordination deficit on the performance of more complex tasks such as steering during locomotion or reaching to targets placed sideways. Future experimental paradigms shall more closely reflect daily life activities, such as shopping at the grocery store (as an example study, see Kim et al., 2020).

## Data Availability Statement

The datasets presented in this study can be found in online repositories. The names of the repository/repositories and accession number(s) can be found below: http://doi.org/10.5281/zenodo.4707477.

## Ethics Statement

The studies involving human participants were reviewed and approved by Comitato etico, ASL 3, Genova. The patients/participants provided their written informed consent to participate in this study. Written informed consent was obtained from the individual(s) for the publication of any potentially identifiable images or data included in this article.

## Author Contributions

DE developed the virtual reality platform and collected and analyzed the data. All authors designed the experiment, wrote and approved the manuscript, contributed to the article, and approved the submitted version.

## Conflict of Interest

The authors declare that the research was conducted in the absence of any commercial or financial relationships that could be construed as a potential conflict of interest.
