# Supplementary material for: Early Blindness Limits the Head-Trunk Coordination Development for Horizontal Reorientation
Source: Front Hum Neurosci. 2021 Jul 16;15:699312. doi: 10.3389/fnhum.2021.699312 (PMC8322610; doi:10.3389/fnhum.2021.699312)
Supplement: Supplementary file 2 [file Data_Sheet_1.docx]

# KINEMATIC SIGNALS PROCESSING

Signals’ nominal sampling rate was 90 Hz. However, Unity’s main loop, which is the one in which sampling happens, does not respect this rate strictly; specifically, the sampling rate is no higher than 90 Hz, but it can be lower. Therefore, when loops take more than 11 ms to complete, some jitter arises in kinematic data. To compensate for such phenomenon, kinematic signals were resampled at 90 Hz. To avoid border artifacts in data resampling, the line passing through first and last data point was subtracted to the signal. Then, resampling was performed using MATLAB’s “resample” function. After resampling, the previously subtracted line was added back. The signal was then smoothed using a 18-samples moving average window. The moving average window length was chosen in order to preserve signal changes with periods longer than 200 ms.

To obtain stationary signals, recurrent differentiation via difference quotients, was performed until all signals tested stationary according to the augmented Dickey-Fuller test, a stationarity test often used in econometrics (Dickey & Fuller, 1979), where time-series analysis is very common. The jerk was the first derivative order where all signals resulted stationary.


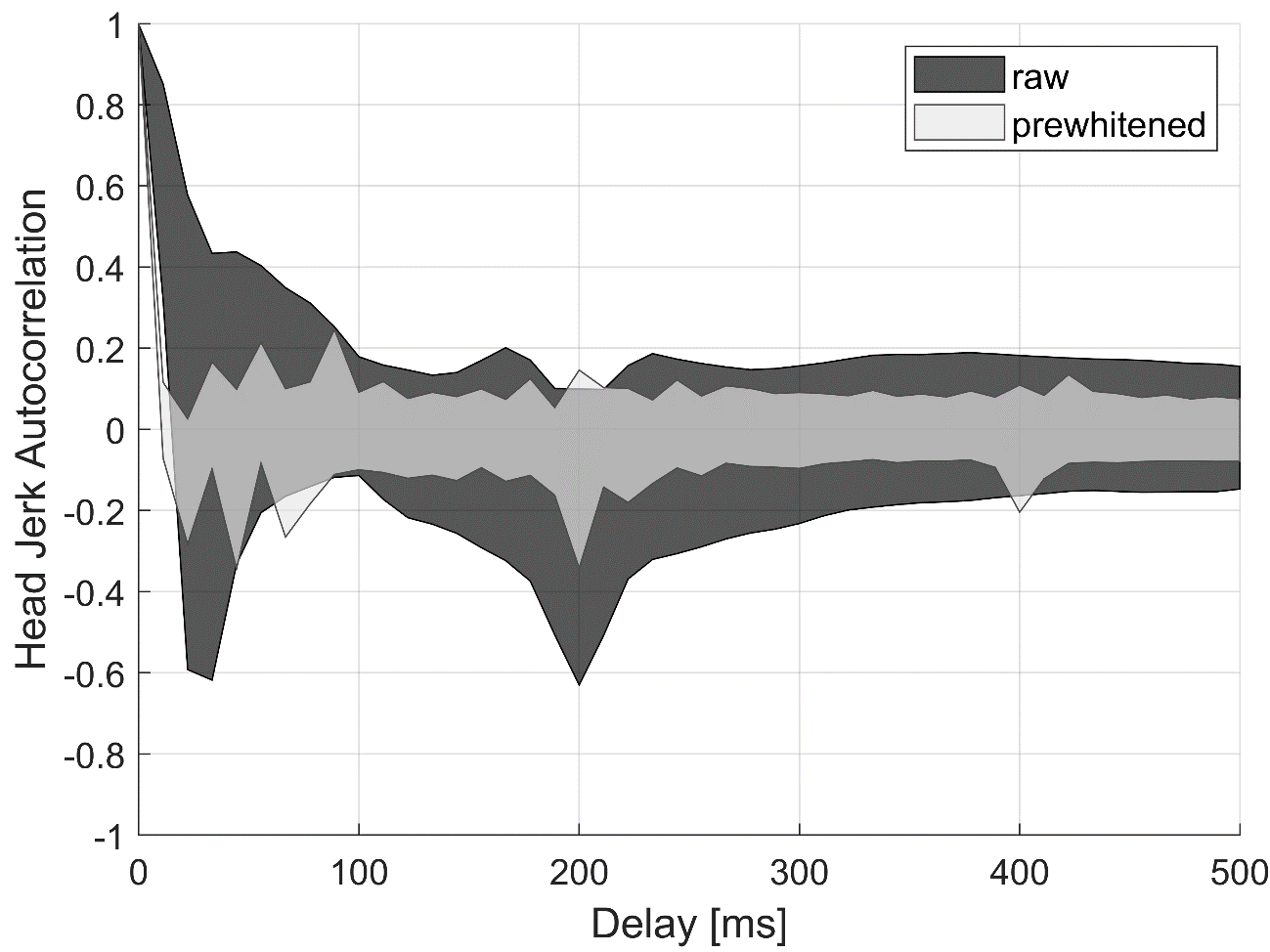


Figure 1s: Lag-wise distributions of correlograms for both raw and prewhitened signals. Filled regions correspond to the distributions’ [-2S, +2S] intervals, where S is the sample standard deviation.

Since kinematic signals do not satisfy the required assumption for cross-correlation analysis of independent, identically distributed (i.i.d.) time-series samples, showing very strong autocorrelation in the first lags, prewhitening was performed using ARIMA modeling to remove autocorrelation from head yaw jerk signals. To prewhiten the time-series in order to perform cross-correlation analysis, the approach based on ARIMA modeling was pursued, as suggested by Dean & Dunsmuir (Dean & Dunsmuir, 2016). This approach consists in: choosing an ARIMA model that well fits one of the two target time-series; taking the model-fitting residuals from the chosen time-series; filtering the other time-series using the chosen ARIMA model as filter. The choice of a proper model order for the given time-series is usually performed by looking at its autocorrelation and partial autocorrelation. However, modeling time-series individually was prohibitive, since our database included 2518 signals. Therefore, the model order selection was based on the observation of the lag-wise distributions of all signals’ autocorrelations and partial autocorrelations, constrained at 45 lags. Based on them, five candidate models were chosen for each time-series: ARIMA(18,1,18) with both AR and MA terms from 5 to 17 locked at 0; ARIMA(4,1,4); ARIMA(18,1,18) with AR terms from 5 to 17 and MA terms from 1 to 17 locked at 0; ARIMA(18,1,1) with AR terms from 5 to 17 locked at 0; ARIMA (4,1,1). The limitation to 5 candidates was a trade-off between model-exploration and temporal demand to perform it. A paired t-test on the distribution of autocorrelation mean squared values, one mean squared value for each lag, before versus after prewhitening, demonstrated the approach to be effective at reducing the signals’ autocorrelation, *t(44)=*4.89, *p*<0.001, 95%CI [0.03,0.08]. Figure 1s shows distributions of both raw and prewhitened autocorrelograms of head yaw jerks for each lag, constrained between [-2S, +2S] where S is the sample standard deviation. To assess the crosscorrelation peak amplitude lag, that gives the temporal relationship between time-series (which series follows the other), prewhitened head jerk autocorrelation peak amplitudes were used as significance thresholds for the corresponding crosscorrelations: if the crosscorrelation peak amplitude was higher than that of the prewhitened head jerk autocorrelation, the null hypothesis of the crosscorrelation value coming from residual autocorrelation is rejected. Figure 2s shows that many crosscorrelation peak amplitudes were below significance threshold. Since peaks were too small to be reliable identifiers of temporal relationship between time-series, the crosscorrelation peak amplitude lag analysis was aborted.


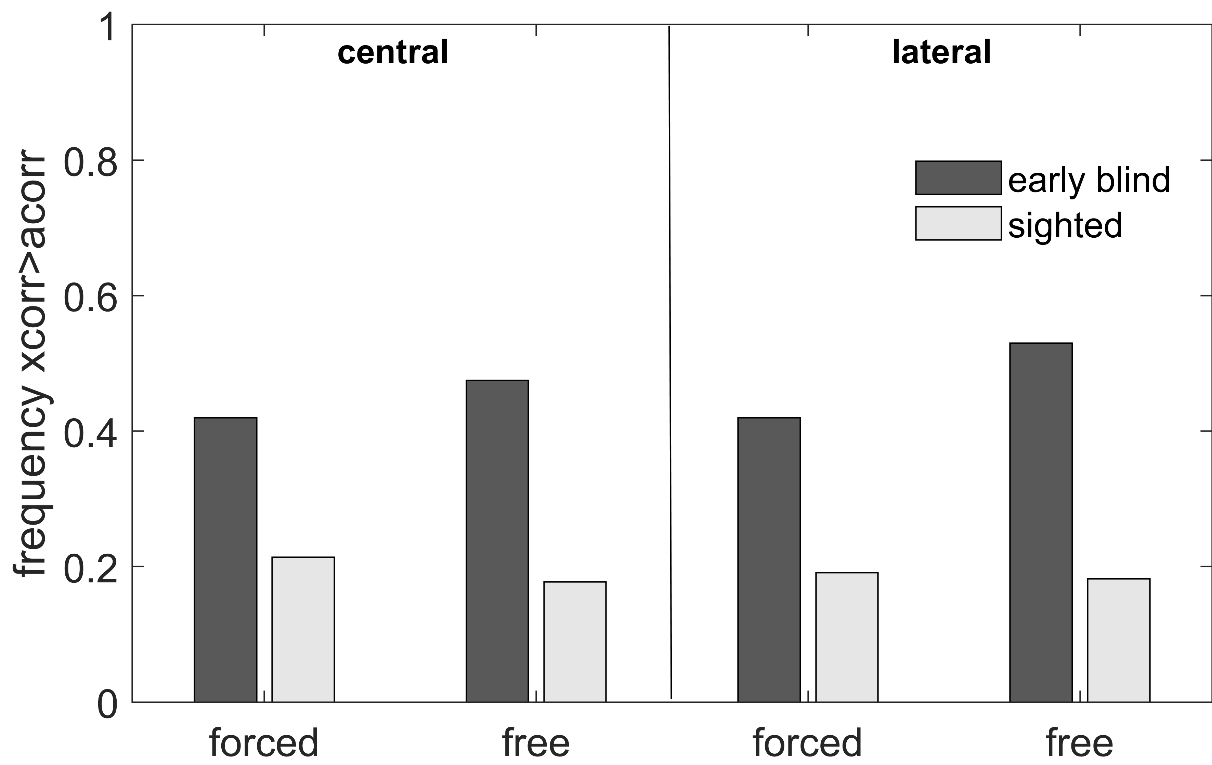


Figure 2s: frequency of trials where the crosscorrelation peak amplitude was higher than the autocorrelation peak amplitude, computed separately for each experimental condition, in both early blind and sighted groups. Low frequencies indicate in many time-series the influence of residual autocorrelation on the peak crosscorrelation cannot be neglected.

# BIBLIOGRAPHY

Dean, R. T., & Dunsmuir, W. T. M. (2016). Dangers and uses of cross-correlation in analyzing time series in perception, performance, movement, and neuroscience: The importance of constructing transfer function autoregressive models. *Behavior Research Methods*, *48*(2), 783–802. https://doi.org/10.3758/s13428-015-0611-2

Dickey, D. A., & Fuller, W. A. (1979). Distribution of the Estimators for Autoregressive Time Series With a Unit Root. *Journal of the American Statistical Association*, *74*(366), 427. https://doi.org/10.2307/2286348
